# Exosomes Derived from Mesenchymal Stem Cells Ameliorate Hypoxia/Reoxygenation-Injured ECs via Transferring MicroRNA-126

**DOI:** 10.1155/2019/2831756

**Published:** 2019-06-02

**Authors:** Qunwen Pan, Yan Wang, Qing Lan, Weiquan Wu, Zhenxuan Li, Xiaotang Ma, Liming Yu

**Affiliations:** ^1^Guangdong Key Laboratory of Age-Related Cardiac and Cerebral Diseases, Institute of Neurology, Affiliated Hospital of Guangdong Medical University, Zhanjiang 524001, China; ^2^Department of Stomatology, Affiliated Hospital of Guangdong Medical University, Zhanjiang 524001, China

## Abstract

Mesenchymal stem cells (MSCs) show protective effects on ischemia/reperfusion- (I/R-) induced endothelial cell (EC) injury and vascular damage. Stem cell-released exosomes (EXs) could modulate target cell functions by delivering their cargos, and exert therapeutic effects as their mother cells. miR-126 is an important regulator of EC functions and angiogenesis. In this study, we determined whether EXs released from MSC-EXs provided beneficial effects on hypoxia/reoxygenation- (H/R-) injured ECs by transferring miR-126. MSCs were transfected with a miR-126 mimic or miR-126 short hairpin RNA to obtain miR-126-overexpressing MSC-EXs (MSC-EXs^miR-126^) and miR-126 knockdown MSC-EXs (MSC-EXs^SimiR-126^). For functional studies, H/R-injured ECs were coincubated with various MSC-EXs. The viability, migration, tube formation ability, and apoptosis of ECs were measured. miR-126 and proangiogenic/growth factor (VEGF, EGF, PDGF, and bFGF) expressions were detected by qRT-PCR. Akt, p-Akt, p-eNOS, and cleaved caspase-3 expressions were examined by western blot. The PI3K inhibitor (LY294002) was used in pathway analysis. We found that overexpression/knockdown of miR-126 increased/decreased the proliferation of MSCs, as well as miR-126 expression in their derived MSC-EXs. MSC-EXs^miR-126^ were more effective in promoting proliferation, migration, and tube formation ability of H/R-injured ECs than MSC-EXs. These effects were associated with the increase in p-Akt/Akt and p-eNOS, which could be abolished by LY294002. Besides, MSC-EXs^miR-126^ were more effective than MSC-EXs in reducing the apoptosis of ECs, coupled with the decrease in cleaved caspase-3. Moreover, compared to MSC-EXs, MSC-EXs^miR-126^ significantly upregulated the level of VEGF, EGF, PDGF, and bFGF in H/R-injured ECs. Downregulation of miR-126 in MSC-EXs inhibited these effects of MSC-EXs. The results suggest that MSC-EXs could enhance the survival and angiogenic function of H/R-injured ECs via delivering miR-126 to ECs and subsequently activate the PI3K/Akt/eNOS pathway, decrease cleaved caspase-3 expression, and increase angiogenic and growth factors.

## 1. Introduction

Vascular endothelial cells (ECs) are critical in maintaining vascular homeostasis [1], and endothelial dysfunction is involved in various ischemic diseases such as limb ischemia, ischemic stroke, and myocardial ischemia [2, 3]. Under ischemia, ECs suffered from hypoxia and reoxygenation (H/R) injury and contribute to the pathogenesis of ischemic diseases [3]. Therefore, understanding EC regulation and protection under H/R injury is pivotal in developing novel preventive and therapeutic strategies for ischemic diseases.

Exosomes (EXs) (30-100 nm) are extracelluar microvesicles that originate from the inward budding of endosomal membranes of cells when activated or during apoptosis [4]. EXs can fuse with cellular plasma membranes of recipient cells and deliver proteins and microRNAs (miRs) into these cells, thereby modulating their functions via various cellular processes and pathways [5, 6]. Recent studies show that stem cell-released exosomes (EXs) contribute a lot to the therapeutic effects of stem cells [7] and have their own advantages [8]. Mesenchymal stem cells (MSCs), which are self-renewing multipotent progenitors that exist in various organs, have showed protective effects on ischemia/reperfusion- (I/R-) induced EC injury and vascular damage [9, 10]. EXs derived from MSCs (MSC-EXs) have been shown to exert therapeutic effects in cardiocerebrovascular diseases and myocardial I/R injury [5, 7, 11]. Recently, MSC-EXs were reported to promote tube formation of normal cultured human umbilical vein ECs [12]. Nevertheless, the effects and mechanisms of MSC-EXs on H/R-injured ECs remain incompletely understood.

miRs, as predominantly functional contents in EXs, play an important role in regulating their functions [6]. A recent study demonstrated that proangiomiRs (e.g., miR-30b and miR-424) in MSCs contributed to the proangiogenic properties of MSC-EXs, which was associated with the regulation of EC angiogenesis [13]. miR-126 is a proangiomiR playing an important role in maintaining vascular homeostasis [14]. miR-126 is enriched in ECs and improves EC proliferation, migration, and angiogenesis [13, 15]. In MSCs, miR-126 can increase the cell survival and secretion of angiogenic factors, which enhanced the therapeutic effects of transplantation of MSCs on functional angiogenesis in the ischemic myocardium [16, 17]. Additionally, miR-126 was detected in stem/progenitor cell-released EXs, and miR-126-overexpressed MSC-EXs can accelerate the angiogenesis in the diabetic rat [18–20]. These indicate that miR-126 might contribute to the effects of MSC-EXs, playing important roles in regulating H/R-injured EC functions.

The PI3K/Akt/eNOS signal pathway and caspase-3 activation have been shown to be involved in EC survival, angiogenesis, and apoptosis processes [21]. Studies showed that miR-126 can regulate the PI3K/Akt/eNOS signal pathway in human cardiac microvascular ECs and human coronary artery ECs [14, 15]. Moreover, numerous studies indicated that miR-126 exerts protective effects on EC proliferation and angiogenesis via regulating growth factors including VEGF, EGF, PDGF, and bFGF [16, 22]. However, whether this pathway and factors are involved in the mechanisms of MSC-EXs and miR-126 that regulate H/R-injured ECs is unknown.

The present study was designed to investigate whether MSC-EXs could exert beneficial effects on H/R-injured ECs through transferring of miR-126. To explore the underlying mechanisms, the PI3K/Akt/eNOS pathway, caspase-3, and growth/angiogenic factors including VEGF, EGF, PDGF, and bFGF were measured.

## 2. Materials and Methods

### 2.1. Cell Culture

Bone marrow was separated from the femurs and tibias of 4 wk old C57BL/6 mice by flushing with culture medium (DMEM; Gibco, USA). The cells were isolated by using the gradient centrifuge method and resuspended in DMEM with 10% fetal bovine serum (FBS, Gibco). Human umbilical vein endothelial cells (HUVECs) were purchased from Shanghai BioLeaf Biotech Co. Ltd. The cells were cultured in DMEM, supplement with 10% FBS in a 37°C incubator with humidified atmosphere of 5% CO_2_/95% air. Cells were digested to conventional passage or cryopreservation when the cells grew to 80% confluence.

### 2.2. Transfection of MSCs

The lentivirus-carrying green fluorescent protein (GFP) marker for gene expression of murine miR-126 mimic (Lv-miR-126-5p), miR-126 silencing short hairpin RNA (Lv-SimiR-126-5p), or scrambled control (Lv-SC) were purchased from GenePharma (Shanghai, China). miR-126-up/downexpressing MSCs were generated as described previously [23]. Briefly, MSCs were transfected with Lv-miR-126, Lv-SimiR-126, or Lv-SC (at 1 × 10^7^infection-forming units) to generate MSC^miR-126^, MSC^SimiR-126^, and MSC^SC^, respectively. The positive cells were observed under a fluorescent microscope, and the transduction efficiency (the expression of miR-126 in MSCs) was quantified by qRT-PCR.

### 2.3. Preparation and Identification of EXs from MSC Culture Medium

The MSCs were cultured in 100 mm plates for 24 h, and then the culture medium was collected and centrifuged at 2000 g for 20 min to remove cells and debris. The collected medium was ultracentrifuged at 20,000 g for 90 min and then ultracentrifuged at 160,000 g for 3 h to pellet MSC-EXs. EXs collected from MSC^SC^, MSC^SimiR-126^, and MSC^miR-126^ were denoted as MSC-EXs, MSC-EXs^SimiR-126^, and MSC-EXs^miR-126^, respectively. The pelleted MSC-EXs were resuspended with filtered phosphate-buffered saline (PBS) and aliquoted for nanoparticle tracking analysis (NTA), transmission electron microscopy (TEM), and flow cytometry analysis [24].

MSC-EXs can be quantified by flow cytometry based on MSC-related surface markers such as CD29 and CD90 [25]. To define MSC-EXs, samples were stained with 5 *μ*L of PE-conjugated anti-mouse CD29 antibody (BD Biosciences) and analyzed by flow cytometry.

Morphology and size of sorted MSC-EXs were further confirmed by TEM, quantified and averaged by examining four random microscopy fields.

### 2.4. Nanoparticle Tracking Analysis

The number and size of MSC-EXs were detected by the NanoSight NS300 instrument as we previously described [21, 24]. In this study, diluted suspensions containing MSC-EXs were loaded into the sample chamber and the camera level was maintained at 9 for light scatter mode. Light scatter mode of NTA used the camera filter 1. Three videos of typically 30-second duration were taken, with a frame rate of 30 frames per second. Data was analyzed by NTA 3.1 software (Malvern Instruments) which was optimized to first identify and then track each particle on a frame-by-frame basis.

### 2.5. Coculture Assay of MSC-EXs with ECs

MSC-EXs were labeled with PKH26 (Sigma-Aldrich, St Louis, MO) according to the manufacturer's protocol with some modifications [24]. Briefly, MSC-EXs were labeled with 2 *μ*M PKH26 at room temperature (RT) for 5 min. An equal volume of 1% bovine serum albumin (BSA) was added to stop staining. MSC-EXs were then ultracentrifuged and resuspended with culture medium. The PKH26-labeled MSC-EXs were added to ECs seeded in glass plates for 24 h incubation (37°C, 5% CO_2_). ECs were stained with actin tracker green (1 : 40, Beyotime) for 1 h. Cell nuclei were then stained with DAPI (1 *μ*g/mL; Wako Pure Chemical Industries Ltd.). The merging of MSC-EXs by ECs was examined under a fluorescence microscope (Leica, TCS SP5II, Germany).

### 2.6. Cell H/R Model

The H/R-induced EC injury model was produced as we previously described [24]. Briefly, the ECs were cultured to 80% confluence in diverse culture dishes, and then they were cultured for 24 h in a hypoxia incubator (Thermo Fisher Scientific, USA) that was equilibrated with 1% O_2_, 5% CO_2_, and 94% N_2_. After that, the cells were preoxygenated by incubation in a standard cell incubator for 24 h. During the reoxygenation time, ECs were cocultured with culture medium (vehicle) or various groups of EXs. ECs cultured with culture medium under normoxic condition were used as controls. Morphological changes of H/R-injured ECs following MSC-EX, MSC-EX^miR-126^, and MSC-EX^SimiR-126^ treatment were observed by an inverted microscope (Life Technologies, USA).

### 2.7. RNA Extraction and Quantitative Real-Time PCR

The levels of miR-126 in MSC, MSC-EX, and MSC-EX-treated ECs were determined. Total miR was extracted by using a miRNeasy Mini Kit (QIAGEN) according to the manufacturer's instructions. The miR-126 cDNA was synthesized using a Hairpin-it™ miR RT-PCR Quantitation Kit (GenePharma, Shanghai, China) using the following parameters: 25°C for 30 min, 42°C for 30 min, and 85°C for 5 min. Real-time PCR parameters were 95°C for 3 min; 40 cycles were performed at 95°C for 12 s and 60°C for 40 s. PCR primers were as follows: 5-TATGGTTGTTCTCGACTCCTTCAC-3 and 5-TCGTCTGTCGTACCGTGAGTAAT-3 for miR-126 and 5-CTCGCT TCGGCAGCACA-3 and 5-AACGCT TCACGAATTTGCGT-3 for U6.

The cDNA of VEGF, EGF, PDGF, and bFGF were synthesized from 1 *μ*g total RNA using the RevertAid First Strand cDNA Synthesis Kit (Thermo Fisher Scientific, USA) at 42°C for 60 min and 70°C for 5 min. Real-time PCR was carried out on a LightCycler 480-II System (Roche Diagnostics, Penzberg, Germany) using SYBR Premix Ex Taq (Takara, Japan) at 95°C for 30 s, 40 cycles (95°C for 5 s, 60°C for 20 s). Each experiment was repeated 3 times. Expression levels were quantified by normalizing the values relative to the mouse housekeeping gene: beta-actin. The relative quantification of the gene expression was determined using the comparative CT method (2^-*ΔΔ*Ct^).

### 2.8. EC Proliferation Analysis

The MTT (3-[4,5-dimethylthiazol-2yl]-2,5-diphenyltetrazolium bromide, 5 mg/mL, Sigma-Aldrich) assay was used to measure cell proliferation. Cells were seeded into 96-well plates at a concentration of 2 × 10^3^ cells/well containing 100 *μ*L of DMEM. After H/R coincubated with a culture medium (vehicle), MSC-EXs, or MSC-EXs^miR-126^ for 24 h, the MTT solution (20 *μ*L) was added and incubated with cells for 4 h at 37°C, and then 150 *μ*L of DMSO was added to each well and incubated with the cells for 20 min at 37°C. The optical density (OD) was read at 490 nm on a microplate reader (BioTek, USA). The percentage of cell proliferation was defined as the relative absorbance of treated cells versus untreated cells. Cells from 3 wells were counted at each time point, and the experiment was repeated 6 times. For pathway exploration, cells were preincubated with PI3K inhibitor (LY294002; 20 *μ*M; Selleckchem) for 2 h [21] and then incubated with MSC-EXs^miR-126^.

### 2.9. EC Migration Analysis

The migration of ECs was measured by scratch assay as we previously described [21, 24]. Briefly, after H/R added with a culture medium (vehicle), MSC-EXs, or MSC-EXs^miR-126^, a scratch was made through the cultured ECs. After 16 h of incubation, the invasion of ECs into the scratched area was observed by an inverted microscope. Quantitative analysis of migration was the percentage of the total cell-free area. Results were calculated from the values obtained in three independent experiments. For pathway exploration, cells were preincubated with PI3K inhibitor (LY294002; 20 *μ*M; Selleckchem) for 2 h [21] and then incubated with MSC-EXs^miR-126^.

### 2.10. EC Tube Formation Analysis

The tube formation ability was measured by using the tube formation assay kit (Chemi-Con) based on manufactory instruments. Briefly, after H/R coincubated with a culture medium (vehicle), MSC-EXs, or MSC-EXs^miR-126^, ECs (1 × 10^4^ cells/well) were placed onto the surface of the EC matrix and incubated with EGM-2 medium (Lonza) for 8 h at 37°C. Five representative fields were taken, and the average of complete tubes formed by ECs in the fields was counted. Results were calculated from the values obtained in three independent experiments. For pathway exploration, cells were preincubated with the PI3K inhibitor (LY294002; 20 *μ*M; Selleckchem) for 2 h [21] and then incubated with MSC-EXs^miR-126^.

### 2.11. Western Blotting

The protein of ECs was extracted with cell lysis buffer (Applygen Technologies Inc., Beijing) supplemented with protease inhibitor tablet (Thermo Fisher Scientific). Protein lysates were electrophoresed through SDS-PAGE gels and transferred onto PVDF membranes. The membranes were blocked with 5% nonfat milk for 1 h and incubated with primary antibodies against beta-actin (1 : 1000, EarthOx, San Francisco, CA, USA), cleaved caspase-3 (1 : 1000, CST, USA), Akt and phospho-Akt (1 : 1000, CST, USA), and phospho-eNOS (1 : 1000, Abcam, USA). Blots were developed with the ECL solution (Amersham, Sweden).

### 2.12. Statistical Analysis

All data were expressed as mean ± SEM. Multiple comparisons were analyzed by one- or two-way ANOVA followed by a Least Significant Distance (LSD) post hoc test. SPSS 23.0 statistical software was used for analyzing the data. For all measurements, a *p* < 0.05 was considered statistically significant.

## 3. Results

### 3.1. Overexpression/Silencing of miR-126 Increased/Decreased the Proliferation of MSCs, as well as miR-126 Expression in MSC-EXs

**Figure 1:**
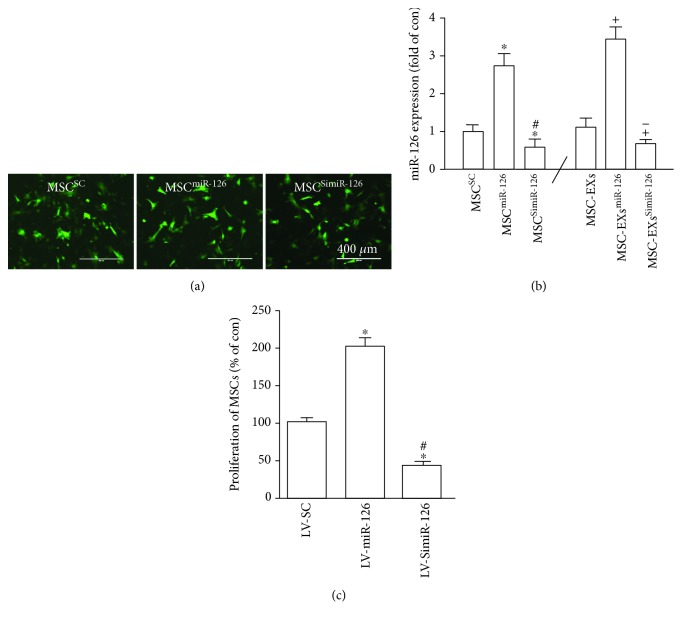
Analysis of the miR-126 expression in MSCs and MSC-EXs, and the effect of miR-126 overexpression/knockdown on the proliferation ability of MSCs. (a) Microscopy images of the GFP marker expression in MSCs after lentivirus infection. Scale bars = 400 *μ*m. (b) Real-time PCR results show the level of miR-126 in MSCs and MSC-EXs. (c) MTT assay of MSC proliferation. ^∗^*p* < 0.05 vs. MSC^SC^; ^#^*p* < 0.05 vs. MSC^miR-126^; ^+^*p* < 0.05 vs. MSC-EXs; ^−^*p* < 0.05 vs. MSC-EXs^miR-126^; *n* = 6/group.

Lv-miR-126 and Lv-SimiR-126 were successfully transfected into MSCs as indicated by the presence of GFP (Figure 1(a)). The efficiency of miR-126 over/downexpression was evaluated by qRT-PCR analysis (Figure 1(a)). The Lv-SC-transfected MSCs (MSC^SC^) and their derived EXs (MSC-EXs) were set as the control. Our results showed that the level of miR-126 in MSCs infected with Lv-miR-126 (MSC^miR-126^) and their derived EXs (MSC-EXs^miR-126^) was significantly increased (vs. MSC^SC^ or MSC-EXs; *p* < 0.05; Figure 1(b)) and decreased in MSC^SimiR-126^ and MSC-EXs^SimiR-126^ (vs. MSC^SC^ or MSC-EXs; *p* < 0.05; Figure 1(b)).

We then used the MTT assay to measure the proliferation of MSCs. We found that Lv-miR-126 markedly increased and Lv-SimiR-126 decreased the proliferation of MSCs (vs. MSC^SC^; *p* < 0.05; Figure 1(c)).

### 3.2. Analyses of Particle Size and Specific Marker Expression of EXs

**Figure 2:**
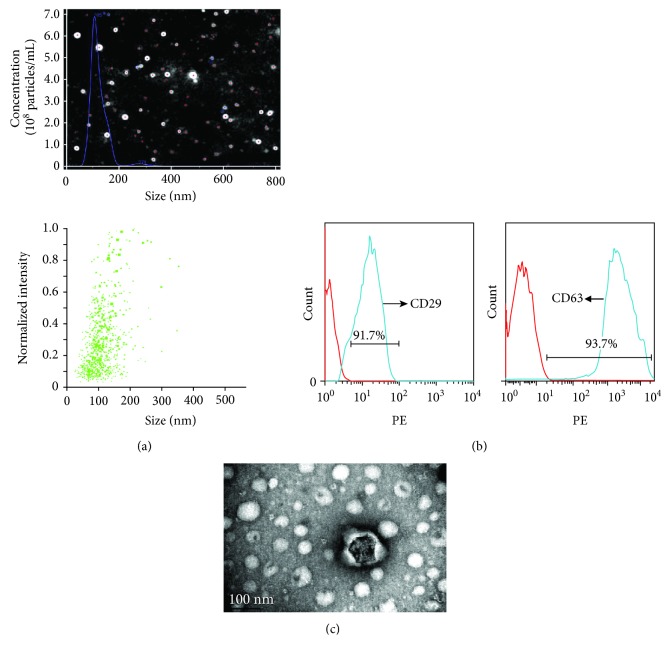
Characterization of MSC-EXs. (a) NTA analysis of MSC-EX size. (b) CD29 and CD63 expressions were determined by flow cytometry. Red line: isotype control; blue line: CD29 positive. (c) Representative image of MSC-EXs examined by TEM.

Flow cytometric analysis showed that MSC-EXs positively expressed the EX marker CD63 (93.7 ± 2.2%, Figure 2(b)) and MSC-specific marker CD29 (91.7 ± 1.5%, Figure 2(b)). Nanoparticle tracking analysis (NTA) and TEM analysis showed that MSC-EXs were in the size of 100 ± 20 nm (Figures 2(a) and 2(c)).

### 3.3. MSC-EX Coincubation Increased the miR-126, VEGF, EGF, PDGF, and bFGF Level in H/R-Injured ECs

**Figure 3:**
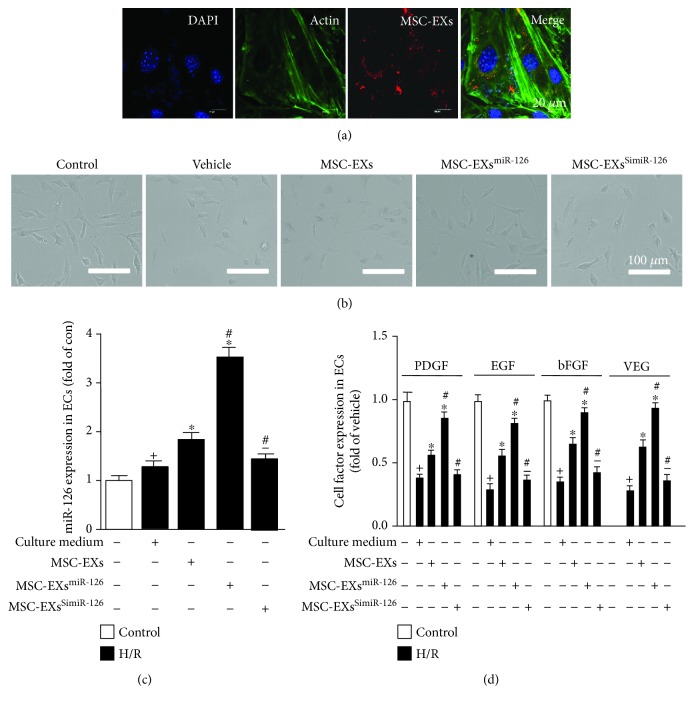
MSC-EX coincubation increased miR-126, VEGF, EGF, PDGF, and bFGF levels in H/R-injured ECs. (a) Representative images showing the incorporation of PKH26-labeled MSC-EXs with H/R-injured ECs after 24 h. Scale bar: 25 *μ*m. (b) Representative images showing the morphological changes of H/R-injured ECs following MSC-EX, MSC-EX^miR-126^, and MSC-EX^SimiR-126^ treatment. (c) Summarized data of the level of miR-126 in ECs. (d) Summarized data of the level of VEGF, EGF, PDGF, and bFGF in ECs. ^+^*p* < 0.05 vs. control; ^∗^*p* < 0.05 vs. vehicle; ^#^*p* < 0.05 vs. MSC-EXs; ^−^*p* < 0.05 vs. MSC-EXs^miR-126^; *n* = 6/group.

We found that PKH26-labeled MSC-EXs could be detected in the cytoplasm of ECs (Figure 3(a)), confirming that MSC-EXs could merge with ECs. After H/R treatment, the ECs shrunk, becoming smaller and round and budded around the cell membrane (*vs*. control; *n* = 6/group; Figure 3(b)). MSC-EXs improved the morphology of the H/R-injured ECs (*vs*. vehicle; *n* = 6/group; Figure 3(b)), and MSC-EXs^miR-126^ were more effective (*vs*. MSC-EXs; *n* = 6/group; Figure 3(b)). The qRT-PCR result showed that the level of miR-126 was significantly increased in MSC-EX-treated ECs (*vs*. vehicle; *p* < 0.05; *n* = 6/group; Figure 3(c)). MSC-EXs^miR-126^ further promoted the miR-126 expression in ECs (*vs*. MSC-EXs; *p* < 0.05; *n* = 6/group; Figure 3(c)), whereas the level of miR-126 was significantly lower in MSC-EX^SimiR-126^-treated ECs (*vs*. MSC-EXs; *p* < 0.05; *n* = 6/group; Figure 3(c)). Moreover, we also found that MSC-EXs significantly promote the cell growth and angiogenic factor (VEGF, EGF, PDGF, and bFGF) expressions (vs. vehicle; *p* < 0.05; *n* = 6/group; Figure 3(d)) in targeted ECs. As expected, MSC-EXs^miR-126^ further promoted VEGF, EGF, PDGF, and bFGF expressions in ECs (vs. MSC-EXs; *p* < 0.05; *n* = 6/group; Figure 3(d)) and MSC-EXs^SimiR-126^ decreased the effects of MSC-EXs (vs. MSC-EXs; *p* < 0.05; *n* = 6/group; Figure 3(d)).

### 3.4. miR-126 Enhanced the Effects of MSC-EXs in Activating the PI3K/Akt/eNOS Signaling Pathway in H/R-Injured ECs

**Figure 4:**
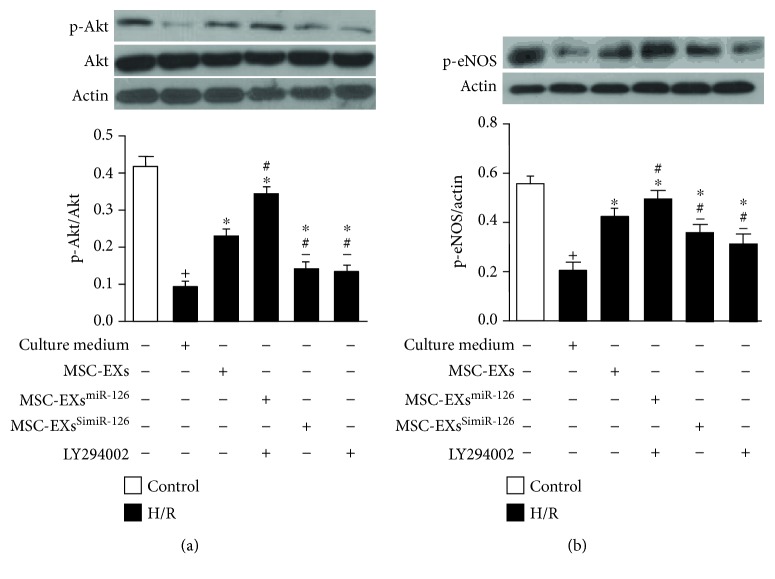
miR-126 enhanced the effects of MSC-EXs in increasing the expression of p-Akt/Akt and p-eNOS in H/R-injured ECs: (a) expression of p-Akt/Akt; (b) expression of p-eNOS. ^+^*p* < 0.05 vs. control; ^∗^*p* < 0.05 vs. vehicle; ^#^*p* < 0.05 vs. MSC-EXs; ^−^*p* < 0.05 vs. MSC-EXs^miR-126^; *n* = 6/group.

To investigate the signal pathways associated with MSC-EXs in protecting H/R-injured ECs, the expressions of Akt, p-Akt, and p-eNOS were analyzed by western blotting. Results showed that H/R decreased the level of p-Akt/Akt, and p-eNOS in ECs (*vs*. control; *p* < 0.05; *n* = 6/group; Figures 4(a) and 4(b)). MSC-EXs increased the levels of p-Akt/Akt and p-eNOS in H/R-treated ECs (*vs*. vehicle; *p* < 0.05; *n* = 6/group; Figures 4(a) and 4(b)). Again, MSC-EXs^miR-126^ were more effective in increasing p-Akt/Akt and p-eNOS expressions in H/R-treated ECs (*vs*. MSC-EXs; *p* < 0.05; *n* = 6/group; Figures 4(a) and 4(b)), and MSC-EXs^SimiR-126^ partially abolished this effect (*vs*. MSC-EXs; *p* < 0.05; *n* = 6/group; Figures 4(a) and 4(b)). Moreover, the effects of MSC-EXs^miR-126^ on p-Akt/Akt and p-eNOS levels were inhibited by PI3K inhibition (*vs*. MSC-EXs^miR-126^; *p* < 0.05; *n* = 6/group; Figures 4(a) and 4(b)). Taken together, these data suggest that miR-126 contributes to the effect of MSC-EXs in promoting EC angiogenic ability via activating the PI3K/Akt/eNOS signaling pathway.

### 3.5. miR-126 Enhanced the Effects of MSC-EXs in Promoting Cell Proliferation and Migration in H/R-Injured ECs by Modulating the PI3K/Akt/eNOS Signaling Pathway

**Figure 5:**
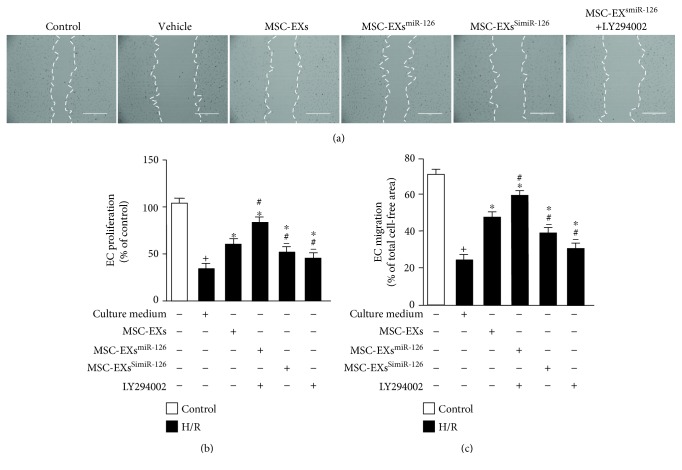
miR-126 enhanced the effects of MSC-EXs in protecting ECs from H/R-induced proliferation and migration dysfunction via the PI3K/Akt/eNOS signaling pathway. (a) Representative image of EC migrations, scale bar: 400 *μ*m. (b) MTT assay of EC proliferation. (c) Summarized data of the migration of ECs. ^+^*p* < 0.05 vs. control; ^∗^*p* < 0.05 vs. vehicle; ^#^*p* < 0.05 vs. MSC-EXs; ^−^*p* < 0.05 vs. MSC-EXs^miR-126^; *n* = 6/group.

According to the MTT assay, we found that H/R decreased EC proliferation (*vs*. control; *p* < 0.05; *n* = 6/group; Figure 5(b)). MSC-EXs increased the proliferation of H/R-treated ECs (*vs*. vehicle; *p* < 0.05; *n* = 6/group; Figure 5(b)). MSC-EXs^miR-126^ were more effective in increasing the proliferation of H/R-injured ECs (*vs*. MSC-EXs; *p* < 0.05; *n* = 6/group; Figure 5(b)), whereas MSC-EXs^SimiR-126^ showed attenuated effect in increasing the proliferation of H/R-injured ECs (*vs*. MSC-EXs; *p* < 0.05; *n* = 6/group; Figure 5(b)). Furthermore, preincubation of ECs with the PI3K inhibitor partially abolished the beneficial effects of MSC-EXs^miR-126^ on EC proliferation (*vs*. MSC-EXs^miR-126^; *p* < 0.05; *n* = 6/group; Figure 5(b)), suggesting that the PI3K signaling pathway contributed to the effects of MSC-EXs on EC proliferation.

The scratch assay analysis results showed that H/R impaired the migration ability of ECs (*vs*. control; *p* < 0.05; *n* = 6/group; Figures 5(a) and 5(c)). MSC-EXs increased the migration of H/R-treated ECs (*vs*. vehicle; *p* < 0.05; *n* = 6/group; Figures 5(a) and 5(c)). MSC-EXs^miR-126^ were more effective in increasing the migration ability of ECs (*vs*. MSC-EXs; *p* < 0.05; *n* = 6/group; Figures 5(a) and 5(c)). As expected, MSC-EXs^SimiR-126^ partially abolished the effects of MSC-EXs (*vs*. MSC-EXs; *p* < 0.05; *n* = 6/group; Figures 5(a) and 5(c)). Again, the PI3K inhibitor partially abolished the beneficial effects of MSC-EXs^miR-126^ on EC migration (*vs*. MSC-EXs^miR-126^; *p* < 0.05; *n* = 6/group; Figures 5(a) and 5(c)). These data indicated that miR-126 enhanced the effects of MSC-EXs on EC proliferation and migration via activating the PI3K signaling pathway.

### 3.6. miR-126 Enhanced the Effects of MSC-EXs in Increasing the Tube Formation Ability of H/R-Injured ECs through Modulation of the PI3K/Akt/eNOS Signaling Pathway

**Figure 6:**
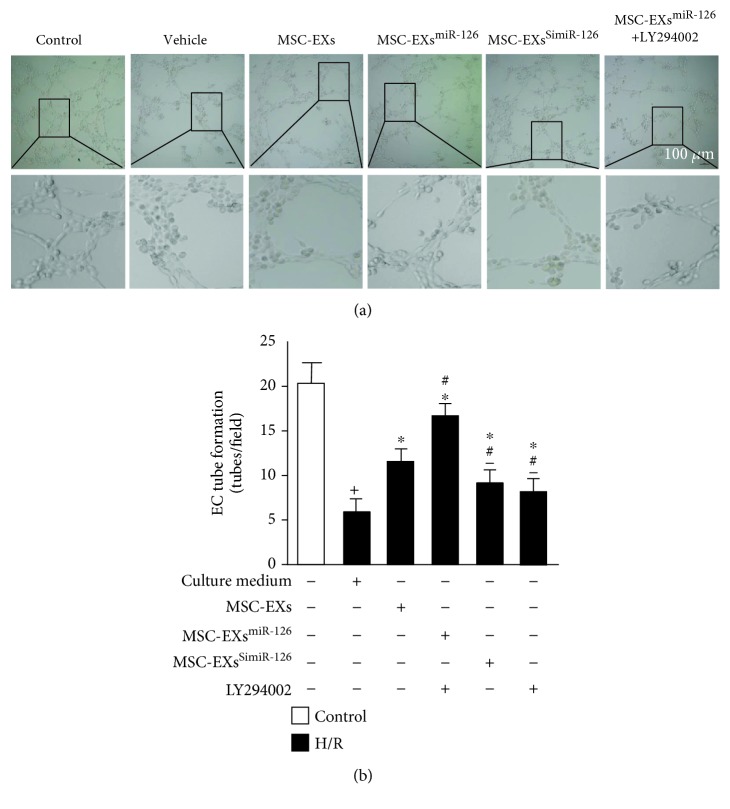
miR-126 enhanced the effects of MSC-EXs in protecting ECs from H/R-induced angiogenic dysfunction via the PI3K/Akt/eNOS pathway. (a) Representative images of EC tube formation. Scale bar: 100 *μ*m. (b) Summarized data on tube formation. ^+^*p* < 0.05 vs. control; ^∗^*p* < 0.05 vs. vehicle; ^#^*p* < 0.05 vs. MSC-EXs; ^−^*p* < 0.05 vs. MSC-EXs^miR-126^; *n* = 6/group.

The effect of MSC-EXs on EC angiogenesis was investigated by an *in vitro* angiogenesis assay. As shown in Figure 6(a), H/R decreased the tube formation ability of ECs (*vs*. control; *p* < 0.05; *n* = 6/group; Figures 6(a) and 6(b)). MSC-EXs increased the tube formation ability of H/R-injured ECs (*vs*. vehicle; *p* < 0.05; *n* = 6/group; Figures 6(a) and 6(b)). Additionally, MSC-EXs^miR-126^ promoted the protective effect of MSC-EXs on the tube formation ability of H/R-injured ECs (*vs*. MSC-EXs; *p* < 0.05; *n* = 6/group; Figures 6(a) and 6(b)). It is not surprising that MSC-EXs^SimiR-126^ partially blocked this effect (*vs*. MSC-EXs; *p* < 0.05; *n* = 6/group; Figures 6(a) and 6(b)), indicating that miR-126 was a proangiogenic functional content in MSC-EXs. Furthermore, preincubation of ECs with the PI3K inhibitor (LY294002) abolished the proangiogenic effect of MSC-EXs^miR-126^ on ECs (*vs*. MSC-EXs^miR-126^; *p* < 0.05; *n* = 6/group; Figures 6(a) and 6(b)), suggesting that the PI3K signaling pathway contributed to the effects of MSC-EXs on EC angiogenesis.

### 3.7. miR-126 in MSC-EXs Decreased the Apoptosis of H/R-Injured ECs Accompanied by the Decreased Cleaved Caspase-3 Level

**Figure 7:**
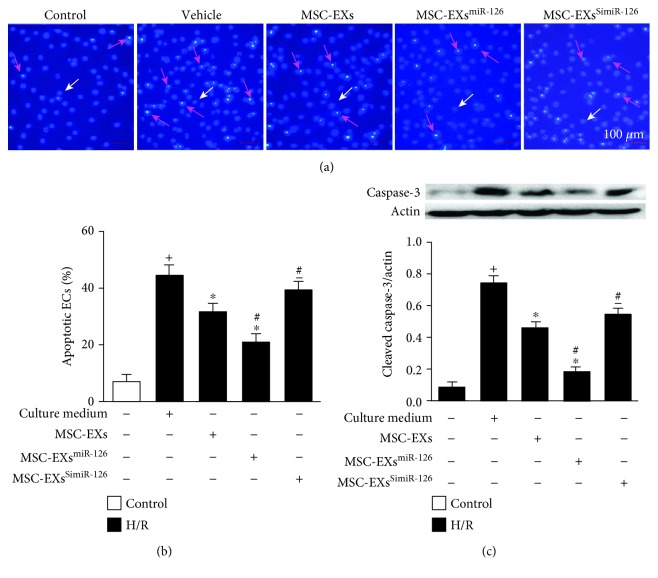
miR-126 enhanced the effects of MSC-EXs in reducing H/R-induced EC apoptosis. (a, b) Representative images and summarized data of EC apoptosis. (c) Cleaved caspase-3 expression in ECs. ^+^*p* < 0.05 vs. control; ^∗^*p* < 0.05 vs. vehicle; ^#^*p* < 0.05 vs. MSC-EXs; ^−^*p* < 0.05 vs. MSC-EXs^miR-126^; *n* = 6/group.

Hoechst 33258 staining revealed that H/R induced apoptosis of ECs (vs. control; *p* < 0.05; *n* = 6/group; Figures 7(a) and 7(b)). MSC-EXs significantly decreased the apoptotic rate of H/R-injured ECs (vs. vehicle; *p* < 0.05; *n* = 6/group; Figures 7(a) and 7(b)). As expected, MSC-EXs^miR-126^ promoted the antiapoptotic effect of MSC-EXs (vs. MSC-EXs; *p* < 0.05; *n* = 6/group; Figures 7(a) and 7(b)), whereas MSC-EXs^SimiR-126^ exhibited a lower antiapoptotic effect on H/R-injured ECs (vs. MSC-EXs; *p* < 0.05; *n* = 6/group; Figures 7(a) and 7(b)).

In addition, we monitored the cleaved caspase-3 which is associated with induction of apoptosis. Results showed that H/R increased the cleaved caspase-3 expression in ECs (vs. control; *p* < 0.05; *n* = 6/group; Figure 7(c)). MSC-EXs decreased the cleaved caspase-3 protein expression in H/R-injured ECs (vs. vehicle; *p* < 0.05; *n* = 6/group; Figure 7(c)). In addition, MSC-EXs^miR-126^ were more effective in decreasing the cleaved caspase-3 expression (vs. MSC-EXs; *p* < 0.05; *n* = 6/group; Figure 7(c)). Again, MSC-EXs^SimiR-126^ partially abolished the effect of MSC-EXs (vs. MSC-EXs; *p* < 0.05; *n* = 6/group; Figure 7(c)).

## 4. Discussion

The present study was set to investigate whether MSC-EXs could promote H/R-injured EC survival and angiogenic function through transferring of miR-126 and explored the underlying mechanisms. We found that miR-126 enhanced the beneficial effects of MSC-EXs on H/R-injured EC proliferation, migration, and tube formation via activating the PI3K/Akt/eNOS signaling pathway and more effectively inhibited H/R-induced EC apoptosis associated with the downregulation of cleaved caspase-3. Meanwhile, miR-126 further promoted the effects of MSC-EXs in elevating the levels of cell growth factors (FGF, bFGF) and angiogenic factors (PDGF, VEGF) in H/R-injured ECs. Downregulating miR-126 partially abolished these effects of MSC-EXs on ECs.

Ischemia/reperfusion- (I/R-) induced endothelial dysfunction is a critical pathological basis for various ischemic diseases [1]. Our group [24] and others [14] have used the H/R condition to model endothelial dysfunction in vitro. In the current study, we reported that H/R-treated ECs displayed impaired proliferation, migration, and tube formation abilities, accompanying increased apoptosis. Our results are well consistent with the previous study which demonstrated that H/R led to EC apoptosis and angiogenic dysfunction [26]. Mesenchymal stem cell- (MSC-) based proangiogenic therapy has been considered as one of the most ideal candidates for the treatment of ischemic diseases [5, 10]. It has been identified that MSCs have the ability to repair the I/R-injured ECs by directly differentiating into functional ECs and/or paracrining a series of growth and angiogenic factors [5, 10]. However, the low survival rate and angiogenic potential of transplanted cells in the ischemic tissue influence the outcome of MSC transplantation for the treatment of ischemic diseases. Recently, several studies have documented that EXs secreted from MSCs (MSC-EXs) play a critical role in MSC-mediated paracrine effects [27, 28]. Moreover, stem cell-released EXs have their own advantage when used in therapy; such as, EXs are small enough to pass through the tissue barrier and they can reduce the potential risks of stem cell therapies, including unwanted engraftment, infusion toxicities due to cell lodging and cellular rejection, and ectopic tissue formation [8, 29]. Thus, to study the effects of MSC-EXs on H/R-injured ECs, we cocultured MSC-EXs with the H/R-injured ECs and found that H/R-induced EC apoptosis was significantly decreased by MSC-EXs. In addition, for the first time, we observed that MSC-EXs significantly ameliorated H/R-injured EC proliferation, migration, and tube formation abilities. These findings are in agreement with a previous report showing that MSC-EXs promoted normal cultured human umbilical vein endothelial cell (HUVEC) tube-like structure formation in vitro [13]. It is well known that the proliferation, migration, and tube formation abilities of ECs contribute to angiogenesis of the ischemic tissue, which is important for the repairment of ischemic diseases including hind limb ischemia, ischemic stroke, and ischemic myocardium [5, 11]. Additionally, EC apoptosis can lead to catastrophic failure of vascular function and homeostasis, which can result in ischemic disease [1]. As we know, at the early stage of ischemic injury, vascular damage is suffered from various pathological factors, such as inflammation, oxidative stress, and hypoxia, leading to EC apoptosis and dysfunction [7]. At the late stage of ischemic diseases, because of the damaged ECs, the angiogenesis and vascular remodeling is impaired in the ischemic tissue [8, 11]. Therefore, our data indicate that MSC-EXs might restore the H/R-injured EC proliferation, migration, and tube formation abilities and prevent EC apoptosis, thus maintaining the vascular homeostasis and function at the early stage of ischemic injury and promoting angiogenesis and vascular remodeling at the late stage of ischemic disease, exerting therapeutic effects on I/R-induced injury.

As we know, miRs are functional contents in EXs and MSC-EXs are rich in miRs (miR-210, miR-132, miR-21, miR-130a, miR-126, etc.) [13, 30]. Reports have demonstrated that stem cell-released EXs showed protective effects on ECs via their carried miRs, such as miR-210, miR-133b, and miR-424 [6, 13, 24]. miR-126 is a key miR in promoting EC angiogenesis and maintaining vascular integrity [14, 31]. Our previous report has shown that EXs derived from endothelial progenitor cells exert protective effects on H/R-injured EC survival and angiogenic function probably due to their carried miR-126 [19]. To investigate whether miR-126 is responsible for the observed effects of MSC-EXs, we examined the role of miR-126-overexpressed/knockdown MSC-EXs in regulating H/R-injured EC functions. Previous studies showed that the contents of EXs could be engineered by modifying the source cells, and miR transfection has been used to modify the composition of the EXs [5, 13]. In this study, we obtained miR-126-over/downexpressing MSC-EXs from miR-126 mimics or inhibitor-transfected MSCs. We found that compared with MSC-EX-cocultured ECs, MSC-EXs^miR-126^ further upregulated the level of miR-126 in ECs, whereas MSC-EXs^SimiR-126^ showed attenuated effect in increasing miR-126 expression. This indicated that miR-126 can be delivered to ECs from MSCs by MSC-EXs. Nevertheless, it is not clear whether the delivered miR-126 could interact with the endogenous miR-126. Of note, it was reported that miR-126 could activate the MAPK/ERK pathway [16] and MAPK/ERK signaling contributes to the upregulation of downstream transcription factor Ets-1 in cancer cells [32]. Moreover, Ets-1 played a key role in controlling the expression of miR-126 in ECs [33]. Thus, we hypothesize that miR-126 delivered by MSC-EXs might increase endogenous miR-126 expression by activating the MAPK/ERK pathway followed with upregulation of Ets-1 in ECs, which needs further investigation in future work. In cell functional analysis, we found that MSC-EXs^miR-126^ showed better effects in rescuing H/R-injured EC survival and angiogenic function. Meanwhile, MSC-EXs^SimiR-126^ exerted attenuated protective effects in H/R-injured ECs. Previous studies have shown that miR-126 was critical for the proangiogenic effect of MSCs on HUVECs and in ischemic myocardium [5, 10, 31]. In the present study, for the first time, we observed that MSC-EXs could protect ECs from H/R injury and verified that miR-126 is an important functional content for the protective effects of MSC-EXs. The enrichment of miR-126 can boost the beneficial effects of MSC-EXs on H/R-induced EC dysfunction. The role of miR-126 in promoting EC proliferation and angiogenic function is consistent with the previous studies [14, 31], Our study reveals a novel role of exosomal miR-126 in MSC-mediated protecting effects on H/R-injured ECs, which provides new insights into the therapy of ischemic diseases with the use of miR-126-rich MSC-EXs.

Numerous studies have demonstrated that the PI3K/Akt/eNOS pathway plays pivotal roles in regulating EC proliferation and angiogenic function [14, 34]. It is reported that miR-126 is implicated in EC and endothelial progenitor cell function by regulating the downstream PI3K/Akt/eNOS signaling pathway [14, 35]. However, it is not clear whether the pathway is implicated in the effects of MSC-EXs on ameliorating H/R-induced EC injury. In the present study, we found that MSC-EXs exerted protective effects on H/R-injured EC angiogenic activities paralleled with the upregulation of Akt and eNOS phosphorylation, indicating that this pathway was involved in the therapeutic effects of MSC-EXs. Moreover, overexpression or knockdown miR-126 in MSC-EXs promoted or attenuated the effects of MSC-EXs on increasing the Akt and eNOS phosphorylation level, and the beneficial effects of MSC-EXs^miR-126^ on H/R-injured ECs could be partially abolished by the PI3K inhibitor. Thus, we demonstrated that MSC-EXs could exert protective effects on H/R-injured ECs through activating the PI3K/Akt/eNOS pathway and miR-126 contributed to the PI3K/Akt/eNOS pathway activation. Our results confirm the role of miR-126 in regulating PI3K/Akt/eNOS as previously reported [14]. Besides, our results showed that MSC-EX^miR-126^-combined PI3K inhibitor treatment could still promote the survival and angiogenic ability of H/R-injured ECs, suggesting that MSC-EXs^miR-126^ might also target other pathways in ECs to ameliorate H/R-induced EC injury, which needs further investigation. Additionally, we also observed that the antiapoptotic effect of MSC-EXs on H/R-injured ECs was linked with downregulation of cleaved caspase-3, an apoptosis-promoting factor [36]. As expected, miR-126 enhanced the effect of MSC-EXs on decreasing the cleaved caspase-3 expression, which is in line with a recent study that demonstrated that miR-126 decreased the apoptosis marker (cleaved caspase-3 and Bcl-2) expression in apoptotic human keratinocytes [37].

It is well known that VEGF, EGF, PDGF, and bFGF are major proangiogenic and growth factors in ECs and the upregulation of these factors promoted the survival and angiogenesis of EC [22, 38]. In this study, to further clarify the regulating mechanism of MSC-EXs, we assessed the VEGF, EGF, PDGF, and bFGF expressions in H/R-injured ECs. We found that MSC-EXs increased the expressions of VEGF, EGF, PDGF, and bFGF in target ECs. Moreover, these effects could be boosted by miR-126 overexpression and attenuated by miR-126 inhibition in MSC-EXs. Our findings add new evidence to the function of miR-126 in regulating cell growth factors and angiogenic factor expressions [16, 39]. Taken together, these data suggested that miR-126 in MSC-EXs not only activated the PI3K/Akt/eNOS signaling pathway but also upregulated the growth and angiogenic factors in H/R-injured ECs. On the other hand, researches have declared that the growth factors and angiogenic factors could activate the downstream PI3K/Akt/eNOS signaling pathway [38]. This provides a possibility that MSC-EXs might exert proangiogenic function by upregulating the growth factors and angiogenic factors and consequently activating the PI3K/Akt/eNOS signaling pathway via transferring of miR-126. Moreover, miR-126 can facilitate VEGF and bFGF signaling, followed by the activated PI3K/Akt/eNOS pathway by directly repressing the target gene expression of the sprouty-related protein 1 (SPRED-1) and phosphoinositol-3 kinase regulatory subunit 2 (PIK3R2) [15, 35]. Another report found that miR-126 could promote CXCR4 expression by inhibiting the regulator of G protein signaling 16 (RGS16) expression [40]. As the target gene of miR-126, CXCR4 play an important role in recruiting EPCs to the ischemic area via activating the downstream PI3K/Akt/eNOS signal pathway [41, 42]. Taken together, miR-126 may upregulate the expression of p-Akt, eNOS, and several growth factors via repressing the expression of the target genes SPRED-1, PIK3R2, and RGS16. However, further studies are needed to confirm the hypothesis, such as knocking down the expression of VEGF, EGF, PDGF, or bFGF, determining the PI3K/Akt/eNOS signaling pathway expression, and carrying out a dual-luciferase reporter assay to confirm the target gene of miR-126. Besides, although we demonstrated the key role of miR-126 in MSC-EXs, we do not exclude that other miRs may also boost the survival and angiogenic capacity of ECs and in vivo studies are needed to verify the therapeutic effect of MSC-EXs in ischemic diseases.

## 5. Conclusion

MSC-EXs have protective effects on ECs against H/R injury including decreasing apoptosis; improving viability, migration, and tube formation ability through miR-126; and subsequently activating the PI3K/Akt/eNOS pathway and increasing cell growth factor (FGF, bFGF) and angiogenic factor (PDGF, VEGF) expressions, while inhibiting the proapoptotic protein cleaved caspase-3 expression.

## Data Availability

The data used to support the findings of this study are included within the article.
